# Progression in multiple sclerosis is associated with low endogenous NCAM

**DOI:** 10.1111/jnc.12236

**Published:** 2013-04-19

**Authors:** Sharmilee Gnanapavan, Peggy Ho, Wendy Heywood, Sam Jackson, Donna Grant, Khadija Rantell, Geoff Keir, Kevin Mills, Lawrence Steinman, Gavin Giovannoni

**Affiliations:** * Department of Neuroimmunology, Institute of NeurologyLondon, UK; † Blizard Institute of Cell and Molecular Science, Barts and The London School of Medicine and Dentistry, Queen Mary University of LondonLondon, UK; ‡ Department of Neurology and Neurological Sciences, Stanford University School of MedicineStanford, California, USA; § Biochemistry Group, Clinical and Molecular Genetics Unit, Institute of Child HealthLondon, UK; ¶ Statistician, Institute of NeurologyLondon, UK

**Keywords:** EAE, mass spectrometry, multiple sclerosis, NCAM, neuroplasticity

## Abstract

Multiple sclerosis (MS) is a CNS disorder characterized by demyelination and neurodegeneration. Although hallmarks of recovery (remyelination and repair) have been documented in early MS, the regenerative capacity of the adult CNS per se remains uncertain with the wide held belief that it is either limited or non-existent. The neural cell adhesion molecule (NCAM) is a cell adhesion molecule that has been widely implicated in axonal outgrowth, guidance and fasciculation. Here, we used *in vitro* and *in vivo* of MS to investigate the role of NCAM in disease progression. We show that in health NCAM levels decrease over time, but this occurs acutely after demyelination and remains reduced in chronic disease. Our findings suggest that depletion of NCAM is one of the factors associated with or possibly responsible for disease progression in MS.

MS is the prototypical inflammatory demyelinating CNS disorder. It has a varied onset and disease course, but progression is the ultimate outcome in the majority of cases. New concepts in pathogenesis have emerged and include theories on axonal degeneration as a prerequisite for progression and remyelination and repair as being important for recovery. Remyelination per se is a frequent finding at the edges of most chronic plaques but absent within its centres (Ozawa *et al*. 1994). Remyelination is a consistent feature in early lesions as evidenced by shadow plaques (Prineas *et al*. 1993). Axonal plasticity, however, is less tangible and accepted dogma is that the adult CNS is unable to spontaneously regenerate after injury (Ramon and Cajal 1989). So why does the endogenous repair process fail in MS?

To be able to recapitulate developmental events for neuronal growth and to enable response to guidance cues, the regenerating axon needs to express the correct complement of cell surface molecules. The neural cell adhesion molecule (NCAM) has been intimately linked to the process of axonal outgrowth, guidance and fasciculation (see reviews by (Gerrow and El-Husseini 2006; Kiryushko *et al*. 2004; Kiss and Muller 2001; Ronn *et al*. 1997). Three major isoforms of NCAM exist in the nervous system: two transmembrane molecules of 140 kDa (NCAM-140) and 180 kDa (NCAM-180), and a third 120 kDa (NCAM-120) glycophosphatidyl-inositol – anchored molecule (Gennarini *et al*. 1984; Nybroe *et al*. 1985). NCAM knockout mice compared with wild-type mice demonstrated a reduction in olfactory bulb size, abnormal fasciculation of hippocampal mossy fibres and a deficiency in long-term potentiation (Tomasiewicz *et al*. 1993; Cremer *et al*. 1994, 1997; Stoenica *et al*. 2006), while double MAG (myelin-associated glycoprotein)/NCAM knockouts demonstrated pronounced axonal pathology in the absence of overt dysmyelination (Loers *et al*. 2004). Isolated NCAM deficiency resulted in pathfinding errors and hypoplasia of the corticospinal tract suggesting a critical role in axonal plasticity (Rolf *et al*. 2002).

Soluble forms of NCAM also exist generated by proteolysis or shedding of whole intact transmembrane forms. They have been identified in neuronal cell culture media, blood and CSF (Bock *et al*. 1987; Krog *et al*. 1992; Olsen *et al*. 1993). Within acutely demyelinated MS plaques paucity of NCAM immunostaining has been demonstrated (Massaro *et al*. 1987), in addition to low levels of circulating NCAM in the CSF, and differential expression of NCAM in active and chronic MS lesions have been identified by proteomics (Han *et al*. 2008). Dynamic measures of NCAM in CSF in MS patients treated with steroids also showed significant elevation paralleling clinical recovery compared with placebo (Massaro 2002).

Here, we investigated the role of NCAM in MS, specifically studying the molecule at molecular, *in-vitro* and *in-vivo* stages. We characterized NCAM in CSF using western blotting and mass-spectrometry. We then studied the temporal expression of NCAM in neuronal aggregates over time and under demyelinating experimental conditions, and validated our findings in acute and chronic experimental autoimmune encephalitis (EAE), and CSF of MS patients.

## Methods

### Materials

Electrophoresis and immunoblotting were preformed using equipment and reagents supplied by Invitrogen, as were materials for the preparation of aggregate cultures. Remaining reagents were purchased from Sigma-Aldrich (St. Louis, MO, USA), unless otherwise specified. Anti-myelin oligodendrocyte protein (MOG) antibody (clone 8-18C5) used for demyelination was from Harlan Sera Lab, Loughborough, UK. Reagent grade double-deionised water and reagents from VWR were used for all proteomic applications. RS100 ProteinChip arrays were from BioRad (Hemel Hempstead, UK), while the Bioprocessor and ProteinChip System and software (version 3.2.1) were from Ciphergen Biosystems, Guildford, UK. Monoclonal anti-human NCAM, polyclonal rabbit NCAM and horseradish peroxidase (HRP)-conjugated swine anti-rabbit antibodies were from BD Biosciences (Oxford, England), Millipore (formerly Chemicon, Watford, UK) and Dako Cytomation (Ely, UK) respectively. Chemiluminescent substrate was purchased from Thermo Scientific (Rockford, IL, USA). NCAM was measured in aggregates and tissues by sandwich ELISA from R & D Systems (Minneapolis, MN, USA) CSF NCAM was measured using a previously described ELISA (Gnanapavan *et al*. 2010). Neurofilament (heavy-chain) and myelin basic protein content were measured using an ELISA as described in (Petzold *et al*. 2003) and (Diemel *et al*. 2004), respectively. Total protein kit was from Bio-rad.

### Western blotting

MS CSF was provided by Neuroimmunology laboratory, National Hospital for Neurology and Neurosurgery, and authorized by the local ethics committee. CSF was centrifuged at 2500 *g* for 10 min and the resulting supernatant was used. Samples were then boiled in LDS sample buffer and reducing reagent, 0.5 M dithiothreitol and electrophoresed on a 4–12% Bis-Tris gel. The gel was electroblotted to nitrocellulose in transfer buffer plus 10% methanol. Non-specific binding was blocked using 2% semi-skimmed milk in saline for 1 h and rinsed off with 0.9% saline. The blot was then incubated overnight at 4°C in primary antibody; anti-NCAM mouse monoclonal antibody diluted 1 : 500 in 0.2% milk. After washing with 0.2% milk in saline containing 0.05% Tween five times at 5 min intervals, the blot was incubated in secondary antibody; swine anti-rabbit-HRP diluted 1/200 in 0.2% milk for 2 h. The washing step was repeated and the HRP activity detected using chemiluminescence.

### Surface-enhanced laser desorption/ionization time of flight mass spectrometry (SELDI-TOF-MS) and protein retrieval

Immunoaffinity capture of NCAM from MS CSF was performed using the eight-spot format RS100 ProteinChip arrays. Monoclonal anti-NCAM antibody containing 1.0 mg/mL of protein in phosphate buffered saline (PBS) was coupled to a single spot on the array (x8) and incubated overnight at 4°C in a humidity chamber. Following this, the residual active sites were blocked using 0.1% bovine serum albumin (BSA)/PBS and incubated for 30 min at 20°C. Unbound antibodies were removed by washing once with 0.1% (v/v) Triton-X PBS wash buffer on an agitator, and twice in PBS (containing no Triton) for 15 min each. The arrays were then placed in parallel in a 96-well format Bioprocessor and 30 μL of crude CSF was added to each spot, while 30 μL 0.2% BSA/PBS was used as control. The Bioprocessor was then placed in a humidity chamber at 4°C and incubated overnight with gentle agitation to facilitate antibody-antigen capture. After incubation, the sample was removed from each array and washed twice for 15 min in wash buffer and once in PBS for 15 min on the agitator. Lastly, to remove the salts, the arrays were rinsed thrice in 5 mM ammonium acetate, pH 7 for 10 s each. The arrays were then air-dried at 20°C. Prior to Surface-enhanced laser desorption/ionization mass spectrometry (SELDI-TOF MS) analysis, 2 × 1 μL saturated sinapinic acid (SPA) matrix in 50% aceto-nitrile (ACN) and 0.1% trifluoroacetic was applied to each spot and air-dried. Mass analysis was performed using the SELDI-TOF ProteinChip System with integrated ProteinChip software collecting the data. Each array was read at high mass with laser intensity set at 288 U, detector sensitivity of 9 and the focus mass optimized from 120 to 180 kDa. Retrieval of antibody-bound protein was carried out prior to the addition of SPA. Using up-and-down motion each spot was rinsed with 3 μL of 70% ACN and 0.2% trifluoroacetic, followed by 3 μL of 50% formic acid: 25% ACN: 15% isopropanol: 10% water. Seven of eight spots were eluted, the last being used as a control. Elute was transferred to an Eppendorf tube and freeze-dried.

### Q-TOF MS analysis

20 μL of 100 mM Tris, pH 7.8 containing 6 M urea was added to the freeze dried sample protein and left to shake at 20°C for 1 h. Disulphide bridges were reduced by the addition of 3 μL of 100 mM Tris-HCL, pH 7.8 containing 5 M DTE and incubation at 20°C for 60 min. Free thiol groups were carboamidomethylated followed by incubation with 6 μL of 100 mM Tris-HCL, pH 7.8 containing 5 M iodoacetamide. The solution was then diluted with 155 μL H_2_O, vortexed and 2 μg of sequence grade trypsin was added to the solution. Samples were incubated overnight at 37°C in a water bath.

Proteins were identified and quantitated by direct analysis of the reaction mixture described above which was spiked with a 1/10 dilution of 1 nmol/mL yeast enolase MassPrep™ digestion standard (Waters Corporation, Manchester, UK). All analyses were performed using a nanoAcquity HPLC and QTOF Premier mass spectrometer (Waters Corporation). Peptides were trapped and desalted prior to reverse phase separation using a Symmetry C18 5 μm, 5 mm × 300 μm pre-column. Peptides were then separated prior to mass spectral analysis using a 15 cm × 75 μm C18 reverse phase analytical column. Peptides were loaded onto the pre-column at a flow rate of 4 μL/min in 0.1% formic acid for a total time of 4 min. Peptides were eluted off the pre-column and separated on the analytical column using a gradient of 3–40% acetonitrile [0.1% formic acid] over a period of 120 min and at a flow rate of 250 nL/min. The column was washed and re-generated at 300 nL/min for 10 min using a 99% acetonitrile [0.1%] rinse. After all non-polar and non-peptide materials were removed the column was re-equilibrated at the initial starting conditions for 20 min. All column temperatures were maintained at 35°C. Mass accuracy was maintained during the run using a lock spray of the peptide [glu1]-fibrinopeptide B delivered through the auxiliary pump of the nanoAcquity (Waters Corporation) at a concentration of 300 fmol/L and at a flow rate of 300 nL/min.

Peptides were analysed in positive ion mode using a Q-Tof Premier mass spectrometer (Waters Corporation), which was operated in v-mode with a typical resolving power of 10 000 FWHM. Prior to analyses, the TOF analyser was calibrated using [glu1]-fibrinopeptide B fragments obtained using a collision energy of 25 eV and over the mass range 50–2000 m/z. Post calibration of data files were corrected using the doubly charged precursor ion of [glu1]-fibrinopeptide B (785.8426 m/z) with a sampling frequency of 30 s. Accurate mass LC-MS data were collected in a data independent and alternating, low and high collision energy mode. Each low/high acquisition was 1.5 s with 0.1 s interscan delay. Low energy data collections were performed at constant collision energy of 4 eV, high collision energy acquisitions were performed using a 15–40 eV ramp over a 1.5 s time period and a complete low/high energy acquisition achieved every 3.2 s.

ProteinLynx GlobalServer version 2.3 (Waters Corporation) was used to process all data acquired. Protein identifications were obtained by searching UniProt/Swiss-Prot databases to which the sequence of P00924 yeast enolase was added manually. Protein identification from the low/high collision spectra for each sample was processed using a hierarchical approach where more than three fragment ions per peptide, seven fragment ions per protein and more than two peptides per protein had to be matched.

### Rat brain aggregate cultures

Serum-free, rotation-mediated neuronal aggregate cell cultures were prepared from foetal Sprague–Dawley rat telencephalon (16 days gestation) as previously described (Diemel *et al*. 2004). Aggregates were then treated with α-MOG plus complement (guinea pig serum) at days 25–29 *in-vitro* (DIV), while control studies received IgG plus complement (IgG control) and complement alone (control). Aggregates were subsequently sampled at various time points (DIV 25–40) and homogenized for analysis of NCAM content using R & D ELISA kit. Total protein content was analysed and NCAM expressed as pg/mg. Neurofilament and MBP were assayed in the control aggregates to assess viability.

### Induction of EAE in SJL and C56BL/6 mice

Female SJL/J and C57BL/6 mice were obtained from The Jackson Laboratory (Bar Harbor, ME, USA) and were immunized at 8–10 weeks of age. All animal protocols were approved by the Division of Comparative Medicine at Stanford University and the Committee of Animal Research at Stanford University School of Medicine, in accordance with the National Institutes of Health guidelines.

SJL/J mice were immunized subcutaneously with 0.1 mg Proteolipid Protein (PLP) 139–151 in PBS emulsified in complete Freund's adjuvant (CFA) consisting of incomplete Freund's adjuvant (Sigma-Aldrich) and 0.4 mg heat-inactivated mycobacterium tuberculosis (strain H37 RA; Difco, Detroit, MI, USA). C57BL/6 mice were immunized subcutaneously with 0.1 mg MOG 35–55 in PBS emulsified in CFA and were boosted intravenously with 400 ng Pertussis Toxin (List Biological Laboratories, Campbell, CA, USA) on days 0 and 2. Animals were examined daily for clinical signs of EAE and scored as follows: Grade 0, healthy; Grade 1, tail paralysis; Grade 2, hind limb paraparesis; Grade 3, hind limb paralysis; Grade 4, complete paralysis (tetraplegy); and grade 5, death. A relapse is defined as an increase in one grade or more in the EAE scale, sustained for at least 2 consecutive days. At the times indicated, mice were killed and perfused with 40 mL ice-cold 1 × PBS before brains and spinal cords were dissected and snap frozen for further analysis.

NCAM concentrations in spinal cords were determined at specific stages in the disease. Spinal cords were mechanically homogenized in T-PER at 1 : 20 (w/v) of tissue to reagent on ice until all visible tissue aggregates had disappeared. Homogenates were then centrifuged at 33 500 *g* for 30 min to remove particulate material, and insoluble material was discarded. To the resulting supernatants protease inhibitors were added at 20 : 1 (w/v).

### CSF NCAM analysis

Sixty-seven MS patient CSF (18 clinically isolated syndrome, CIS, 29 relapsing-remitting multiple sclerosis, RRMS, 14 secondary progressive multiple sclerosis, SPMS, six primary progressive multiple sclerosis; 43 females and 24 males; mean age 42.4 ± 12.1, range 22–64 years) were included in the study. Detailed clinical data were available on 64 of 67 patients; 25 samples were taken during an MS relapse, and expanded disability status scale (EDSS) scores ranged from 0 to 7, none of the patients received corticosteroids during the course of sampling. NCAM levels were measured using a previously validated in-house ELISA (Gnanapavan *et al*. 2010). Briefly, 100 μL 2.5 μg/mL of mouse monoclonal anti-NCAM (capture antibody) in 0.01 M PBS was coated overnight at 4°C in polystyrene wells. Wells were washed once with 0.05% (v/v) Tween/PBS and blocked with 5% semi-skimmed milk for 1 h at 20°C on a shaker. Wash step was repeated but the wash solution was left to stand for 5 min before decanting. Aliquots of 25 μL standards, controls and CSF samples to 75 μL 5% semi-skimmed milk in PBS wash solution were added to duplicate wells and incubated overnight at 4°C with shaking. The plate was washed five times leaving to stand 5 min per wash in PBS wash solution, and then coated with 100 μL of polyclonal rabbit NCAM (detector antibody) at 1/1000 dilution for 1 h with shaking. Washing step was repeated again for five washes and then 100 μL of HRP-conjugated swine anti-rabbit (reporter antibody) was added to each well. Plate was incubated on the shaker for further 1 h before being washed as stated earlier. Lastly, the plate was developed using 100 μL TMB (supersensitive 3,3′, 5,5′ tetramethylbenzidine) and stopped with 50 μl 1 M HCL. Absorbencies were measured photometrically at 450 nm (test) and 750 nm (blank) and NCAM concentrations calculated using the standard curve.

### Statistical analyses

All analyses were performed using SPSS (version 15, New York, NY, USA) statistical software. Before proceeding, the data were assessed for normality using the Shapiro–Wilk test. Differences between means were calculated using one-way anova with Bonferroni correction for multiple comparisons. For the CSF data, we used a univariate general linear model to evaluate the influence of variables age and sex on the dependent variable NCAM. Mean difference in NCAM between acute (relapse) and non-acute samples were evaluated by independent *t*-test. We compared NCAM values and EDSS scores by Sperman's rank correlation to look for trends. Logistic regression modelling was used to analyse the associations between NCAM and EDSS severity, EDSS was dichotomized into mild (EDSS ≤ 3) and severe (EDSS > 3), with severe EDSS as the reference category. EAE analysis was performed using GraphPad Prism (version 5, San Diego, CA, USA) software, with Kruskal–Wallis test and Dunn's *post hoc* evaluation.

## Results

### Identification of soluble NCAM in CSF

**Fig. 1:**
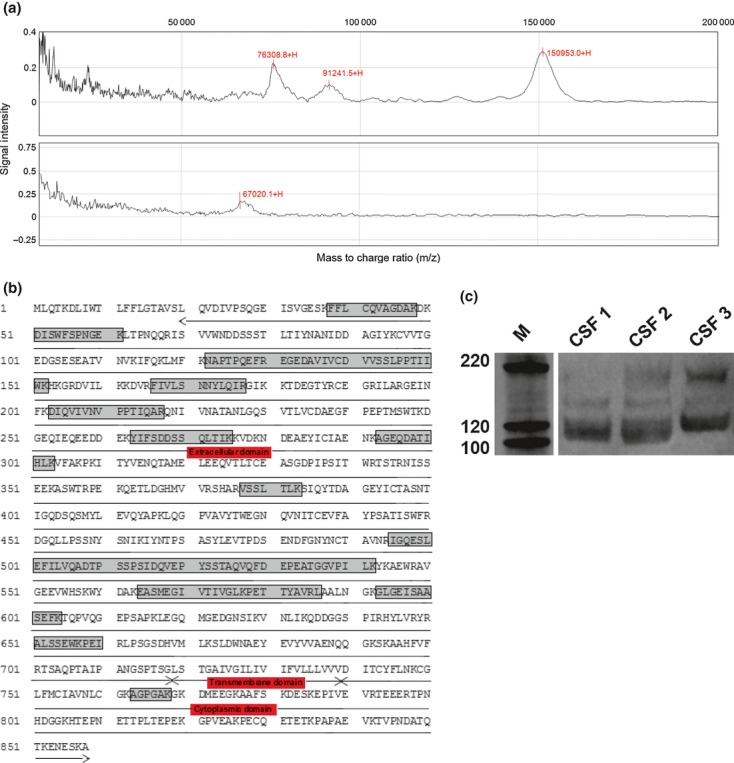
(a) Representative Surface-enhanced laser desorption/ionization-TOF mass spectrometry (MS) spectra from an MS patient (top) with a control experiment with 0.2% bovine serum albumin only (bottom); *m/z* ratio of the peaks are annotated; (b) ESI Q-TOF sequence mapping for CSF neural cell adhesion molecule (NCAM), the peptide data (noted by the boxed areas) mark sites of coverage on the NCAM sequence and the topological domains (annotated by arrows); and (c) western blot analysis of NCAM in the CSF of three MS patients showing the three major isoforms.

Protein profiling by antibody pull down and SELDI MS revealed distinct cluster of peaks at mass to charge ratio (*m/z*) of approximately 75 kDa, 91 kDa, 127 kDa, 137 kDa, and 150 kDa in MS CSF. Fig. 1a, shows a representative profile from an MS patient (top) and a control run using 0.2% BSA. Characterization of protein retrieved from the chips by ESIQ-TOF MS corresponded to sequence motifs from the extracellular Ig-like C2-type 1-5 domains, fibronectin type-III 1, 2 domains, and the intracellular cytoplasmic domain of NCAM (Fig. 1b). This suggests that it is possible to have the entire length of NCAM polypeptide in the CSF. Further analysis by western blot showed three polypeptide bands with an apparent molecular range of 120–200 kDa, with 120 kDa band being predominant. Their relative proximity to each other suggests that they are isoforms of NCAM (Fig. 1c).

### Differential modulation of NCAM during development and following demyelination

**Fig. 2:**
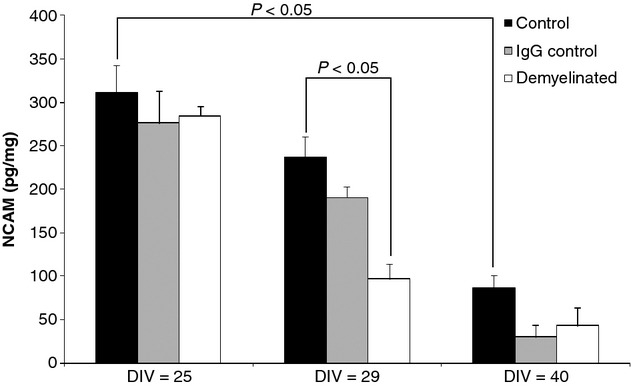
Neural cell adhesion molecule (pg/mg, mean ± SE) in rat neuronal aggregate cultures at day *in vitro* 25, 29 and 40 and following demyelination with α-MOG. Significant *p*-values are shown

In neuronal aggregates, NCAM concentrations decreased in all three experimental groups over the lifespan of aggregate cultures suggesting a developmental reduction in the protein, a finding which is consistent with its role in axonal growth and plasticity (Fig. 2). Introduction of the demyelinating agent α-MOG at the optimum age of the cultures (DIV 25) resulted in a significant reduction in NCAM afterwards (Fig. 2). By DIV 40, NCAM levels fell below the level of detection of the assay in both the α-MOG and control experiments. Neurofilament content was measured at 109.1 μg/mg ± 8.1 (DIV 29) and 111.8 μg/mg ± 3.0 (DIV 40), while MBP was measured at 5.8 μg/mg ± 1.7 (DIV 29) and 8.4 μg/mg ± 3.4 (DIV 40) in the controls.

### Persistent reduction of NCAM in chronic EAE

**Fig. 3:**
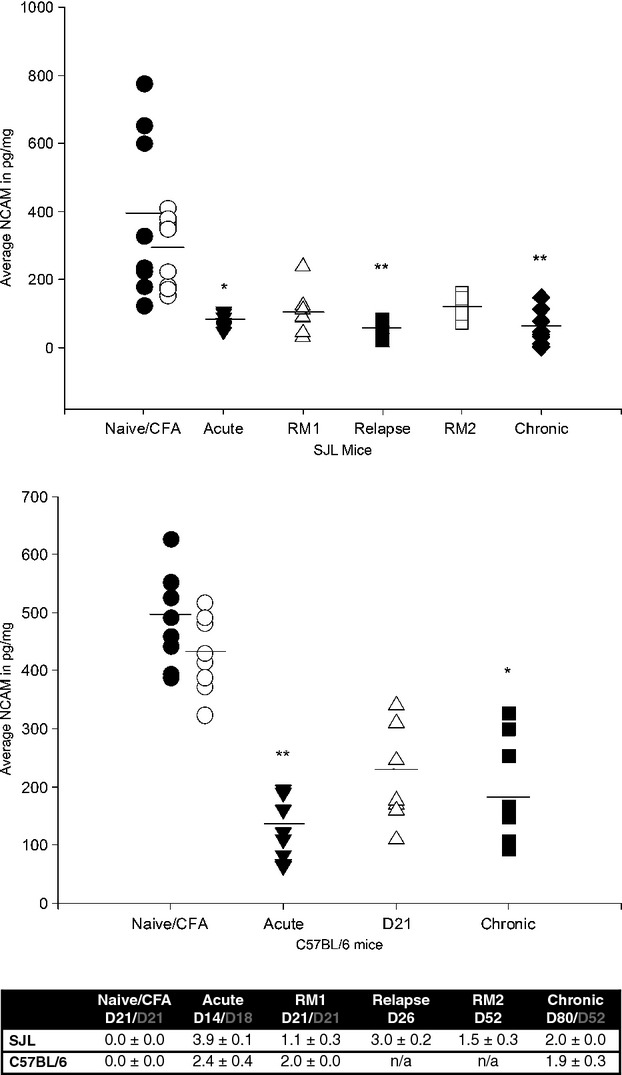
Neural cell adhesion molecule (NCAM) (pg/mg) in relapsing-remitting (SJL) and chronic (C57BL/6) experimental autoimmune encephalitis (EAE). Mean difference is significant at 0.05 (*) and 0.001 (**) level after Dunn's *post hoc* test. The line denotes mean NCAM content. The table demonstrates the clinical disease scores of EAE in SJL and C57BL/6 mice during the different phases of EAE. EAE was induced in mice on day 0. The results represent the mean clinical score ± SEM during active disease or the minimal score during remission when tissue was sampled for NCAM-specific ELISA (*n* ≥ 8). The days of sampling are indicated in white for SJL mice and grey for C57BL/6 mice. Complete freund's adjuvant. RM1 = remission 1. RM2 = remission 2.

NCAM was investigated in SJL and B6 mice which exhibit a relapsing-remitting disease course and a chronic course in EAE respectively (Fig. 3). There was a significant drop in NCAM levels in acute EAE at the peak of the disease in both models compared with naïve animals (Naive/CFA vs. acute relapse *p* < 0.05, 1st relapse *p* < 0.001 in SJL mice and Naive vs. acute relapse *p* < 0.001 in B6 mice). Similarly, during the chronic phase of the disease NCAM levels remained low in both models with no recovery in NCAM levels by this point (Naive/CFA vs. chronic phase *p* < 0.001 in SJL mice and Naive *vs*. chronic phase *p* < 0.05 in B6 mice). Although not significant, during remission stages in the spinal cords of the SJL mice, an upward trend in mean NCAM levels was apparent.

### Soluble NCAM levels vary between different disease subtypes

**Fig. 4:**
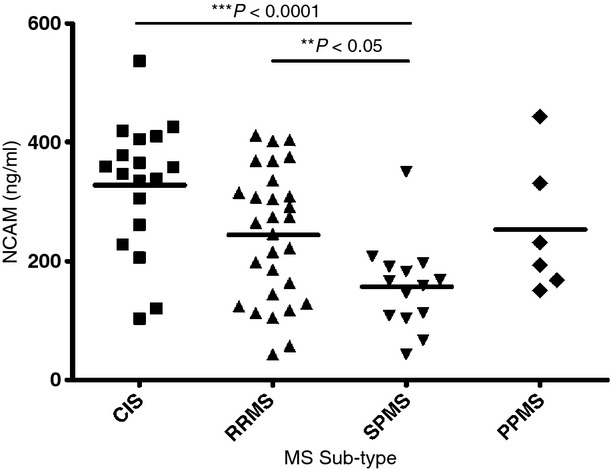
Neural cell adhesion molecule (ng/mL) in the CSF of MS subtypes (CIS, clinically isolated syndrome; RRMS, relapsing-remitting MS; SPMS, secondary progressive MS; PPMS, primary progressive MS). The horizontal bars represent the mean for each group. Significant *p*-values are shown.

**Fig. 5:**
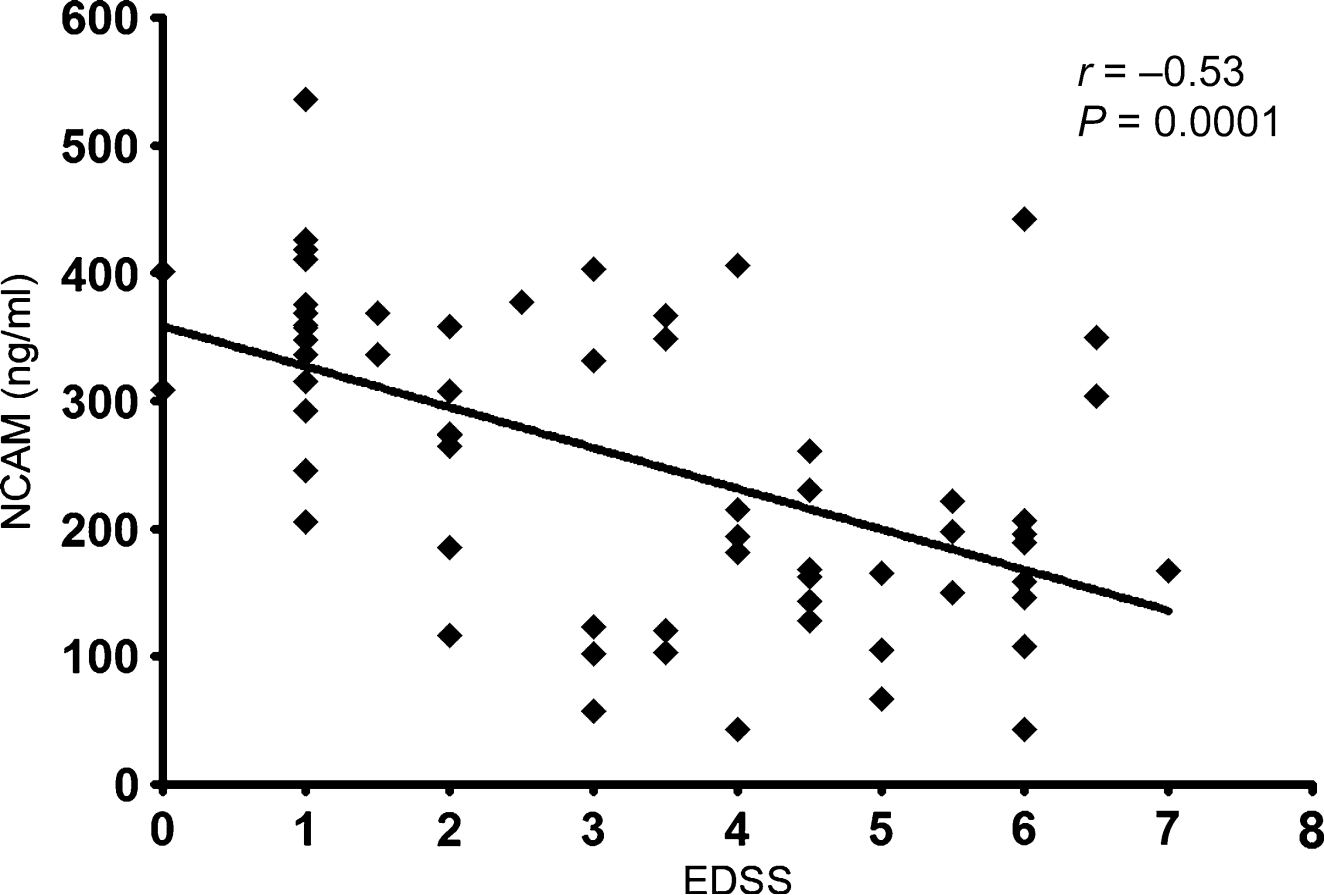
Spearman rank correlation relating CSF Neural cell adhesion molecule (ng/mL) to EDSS. The correlation coefficient (rho) and *p*-value are shown.

The antibody which recognizes the amino-terminus that is shared by NCAM-120, −140 and −180 was used to quantify the levels of soluble NCAM in MS CSF (same antibody used for western blotting). NCAM levels were found to be reduced in a step-wise manner in the order of CIS > RRMS > SPMS (Fig. 4). Statistically significant reductions were found between CIS and SPMS (*p* < 0.0001) and RRMS and CIS (*p* = 0.031). No significant differences were found between primary progressive multiple sclerosis and the others. A Spearman's rank correlation that NCAM was negatively correlated with the EDSS (*r* = −0.53, *p* = 0.0001) (Fig. 5). Differences in NCAM levels were found to be unrelated to either age or gender. Through logistic regression, NCAM was found to be significantly associated with EDSS, with every unit decrease in NCAM increases the odds of being in the severe disease severity category by 1% (OR 1.01, 95% CI 1.004–1.015, *p* < 0.0001). NCAM levels were slightly higher in the non-acute samples by 26 ng/mL but not significant (95% CI −87–34 ng/mL, *p* = 0.39).

## Discussion

The renewed focus on neuroregenerative strategies in MS have highlighted considerable gaps in our understanding of CNS repair and plasticity, and why cellular biology increasingly matters. Here, we present data to suggest cell adhesion molecule NCAM which is critical for axonal regrowth and neuronal migration is irretrievably damaged in the injured CNS both *in-vitro* and *in-vivo*. Moreover, this effect may be amplified by the temporal down-regulation in endogenous NCAM with ageing. Both are important considerations for future neuroreparative work.

NCAM-120 is the predominant isoform in the CSF probably owing to its largely extracellular configuration (Bock *et al*. 1987; Krog *et al*. 1992; Poltorak *et al*. 1996), even though based on our sequence analysis both the extracellular protein fragment, as well as a small portion of the cytoplasmic fragment can be found in the CSF. In support, work on human pre-frontal cortex demonstrated ectodomain shedding of an extracellular 105–115 kDa fragment (NCAM-EC) and a smaller 30 kDa intracellular fragment (NCAM-IC), thought to be derived from the cytoplasmic domain of NCAM-140 (Cox *et al*. 2009). Soluble forms of NCAM have been observed in culture media and body fluids, though their precise role is unclear (Ibsen *et al*. 1983; Bock *et al*. 1987; Krog *et al*. 1992). It is postulated that soluble NCAM interferes with homophilic adhesion between membrane bound NCAM resulting in de-adhesion and modulation of NCAM dependent neuronal growth. Specific inhibition of NCAM release from the membrane by blocking metalloproteinase cleavage (using inhibitors BB-3103 or GM 6001) resulted in aggregation of neurones in primary hippocampal neurones in culture and NCAM-140 expressing B35 neuroblastoma cells (Diestel *et al*. 2005; Hubschmann *et al*. 2005).

Our study on neuronal aggregates demonstrates a temporally precise modulation of NCAM expression. Although the expression persists into the adult stage beyond developmental expression, it is at a fraction of the original expression. We did not find a similar drop in MBP or neurofilament content of the aggregates. This has obvious implications for enhancing repair in the ageing CNS. Antibody-mediated demyelination results in a marked reduction in NCAM suggesting acute axonal damage. We have previously shown that demyelination induces immunopositivity to SMI32 in aggregates, i.e. changes the phosphorylation status of the axon (Jackson *et al*. 2004). In EAE, a T-cell mediated inflammatory model, the drop in NCAM is immediate and persists into the chronic stages suggesting that the end-result is identical despite the differences in aetiopathogenesis. Moreover, there is no definite recovery in NCAM levels during periods of disease remission suggesting that the precise point in the temporal decline of NCAM is reached much sooner. Correspondingly, soluble NCAM levels in MS patients decreases in a step-wise manner through the various stages of disease, starting from first presentation and progressing towards fixed disability, and there's no appreciable difference in NCAM levels with relapses. Unlike the aggregates we did not observe a corresponding decline in CSF NCAM levels with age. We found a moderate negative correlation between NCAM levels and EDSS disease severity, with decreasing NCAM levels being associated with more severe disability. Evidence from natural history studies in MS suggest that this progression occurs during the sixth decade of life in most individuals and is unaffected by the initial course of MS, whether relapsing-remitting or progressive (Confavreux and Vukusic 2006; Kremenchutzky *et al*. 2006). This is supported by work in the SJL mice which exhibit a relapsing-remitting course of EAE compared to B6 mice which have a more chronic disease course, wherein the reduction in NCAM levels is the same despite clear difference in disease pathophysiology. In either case, the only constant is the insult to the CNS.

Interestingly, with soluble NCAM we found considerable overlap between the RRMS and SPMS patients which may be a sign of the functional limitations of the clinical classification system. Previous groups have reported low NCAM levels in the CSF and are thought to represent a lowered potential for endogenous repair (Massaro 2002; Strekalova *et al*. 2006). Evidence from NCAM deficient mice suggests that NCAM plays a critical role in the development of the corticospinal tract, wherein deficiency leads to profound pathfinding errors and hypoplasia (Rolf *et al*. 2002). It is then not surprising that NCAM deficiency can result in profound disability and may contribute to the onset of progression in MS. Steroid treatment after an acute exacerbation appears to lead to a rise in CSF NCAM, suggesting that it may be possible to stimulate endogenous NCAM levels therapeutically (Massaro 2002).

In summary, our study on NCAM suggests disability progression in MS may be a direct or an indirect consequence of NCAM deficiency. Our results indicate that there is a developmental down-regulation of NCAM over time and demyelination can adversely impact on axonal plasticity. Moreover, demyelinating events directly cause reduction in NACM levels, events which impact on recovery and disease progression in MS. It remains to be seen whether stimulating NCAM production can successfully recapitulate developmental cues for neuronal growth.

## Abbreviations

ACN: aceto-nitrile
BSA: bovine serum albumin
CFA: complete Freund's adjuvant
CIS: clinically isolated syndrome
EAE: experimental autoimmune encephalitis
HRP: horseradish peroxidase
MS: multiple sclerosis
NCAM: neural cell adhesion molecule
PBS: phosphate buffered saline
RRMS: relapsing-remitting multiple sclerosis
SELDI: surface-enhanced laser desorption/ionization mass
SPA: sinapinic acid
